# Progress in the Applications of Smart Piezoelectric Materials for Medical Devices

**DOI:** 10.3390/polym12112754

**Published:** 2020-11-22

**Authors:** Angelika Zaszczyńska, Arkadiusz Gradys, Paweł Sajkiewicz

**Affiliations:** Institute of Fundamental Technological Research, Polish Academy of Sciences, Pawinskiego 5b St., 02-106 Warsaw, Poland; argrad@ippt.pan.pl (A.G.); psajk@ippt.pan.pl (P.S.)

**Keywords:** polymers, smart materials, piezoelectric materials, inorganic materials, organic materials, biomedical devices

## Abstract

Smart piezoelectric materials are of great interest due to their unique properties. Piezoelectric materials can transform mechanical energy into electricity and vice versa. There are mono and polycrystals (piezoceramics), polymers, and composites in the group of piezoelectric materials. Recent years show progress in the applications of piezoelectric materials in biomedical devices due to their biocompatibility and biodegradability. Medical devices such as actuators and sensors, energy harvesting devices, and active scaffolds for neural tissue engineering are continually explored. Sensors and actuators from piezoelectric materials can convert flow rate, pressure, etc., to generate energy or consume it. This paper consists of using smart materials to design medical devices and provide a greater understanding of the piezoelectric effect in the medical industry presently. A greater understanding of piezoelectricity is necessary regarding the future development and industry challenges.

## 1. Introduction

Biomaterials, including those indicating piezoelectric effect, are a group of synthetic or natural materials which can communicate effectively with biological structures for the therapeutic or diagnostic purpose [1]. Considering such applications as tissue engineering [2,3], minimally invasive sensors [4,5,6], actuators [7,8] drug delivery systems [9], energy harvesting [10,11,12], storage [13], etc. [14], biomaterials should be biocompatible (nonimmunogenic), non-injurious, and nontoxic. Piezoelectric materials are a class of inorganic and organic materials (mainly polymers) that can transform electricity into mechanical force and vice versa. In crystals, piezoelectricity occurs along with the ions in the structures of dielectric materials [15]. Polarization of the materials changes linearly with applied force, causing the electrical field in the material. In organic materials, especially piezoelectric polymers, the piezoelectric effect is invoked by the orientation and the molecular structure of the piezoelectric polymer [16].

Piezoelectricity occurs in different mammalian tissues consisting of α-keratin with aligned α-helical structures such as wool, hair, hooves, and horns. A lot of elements of the muscles and skeletal tissues have a collagen structure. Collagen is characterized by helical fibrils and spiral structures. Along the fibril axis, every collagen fibril exhibit a lateral piezoresponse. Consequently, many tissues in nature are piezoelectric e.g., bones, ligaments, cartilage, and tendons.

**Figure 1:**
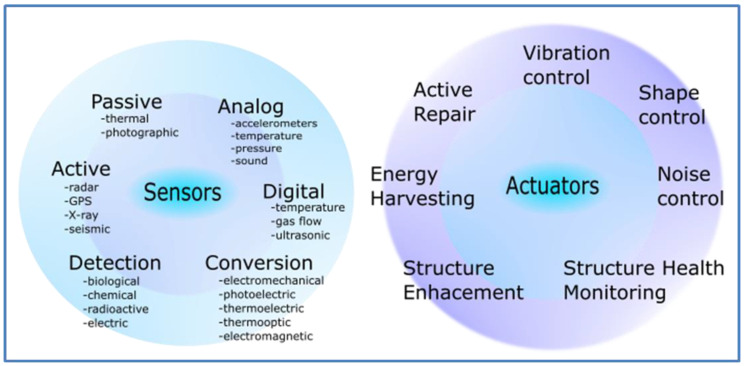
Applications of actuators and sensors.

The global demand for piezoelectric medical devices is valued at approximately 20 billion euros per year, with a large share of piezoelectric sensors and actuators. Taking advantage of the mechanical energy to support small scale devices is possible [17]. Piezoelectric applications, which include interfaces with biological structures, represent a pioneering rapid development [17,18]. Actuators and sensors also play a significant role in different practical applications [19,20], what is illustrated in Figure 1.

**Figure 2:**
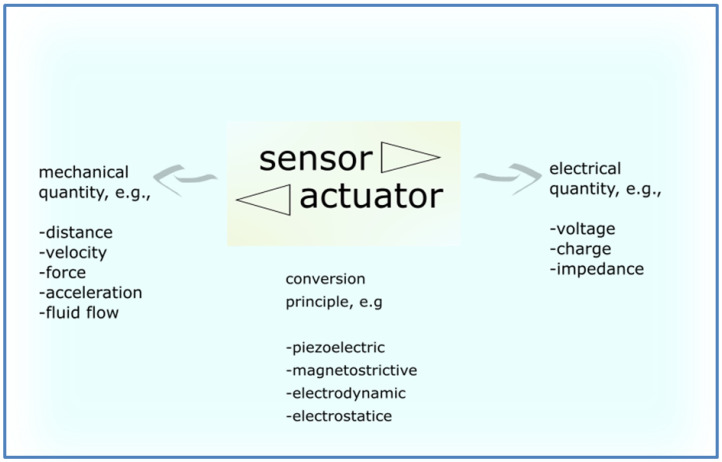
Actuators and sensors—a typical transformation of selected quantities.

In general, sensors transform mechanical energy into electrical energy. Figure 2 shows typical transformations of various quantities. Sensors can convert e.g., mechanical force to an electric voltage. Reverse to sensors, actuators transform electrical quantities (e.g., electric voltage) into mechanical measurands (e.g., mechanical force). Electrical quantities constitute the inputs, where mechanical energy represent the outputs of actuators [21].

Inorganic piezoelectric materials might be natively biocompatible e.g., barium titanate (BaTiO_3_) [22], quartz [23], or can be biocompatibilized through processing—this includes materials such as aluminum nitride (AlN) [24], lithium niobate (LiNbO_3_) [25], zirconate titanate (PZT) [26] and zinc oxide (ZnO) [27]. Pressure tuning, ultrashort laser pulses, or microwaves are used to increase the biocompatibility of inorganic piezoelectric materials.

Organic polymers, e.g., polyvinylidene fluoride (PVDF), are piezoelectric and ferroelectric after additional treatment, particularly after poling [28]. Poly-d-lactic acid (PDLA) and poly-l-lactic acid (PLLA) are a group of optically active polymers showing piezoelectricity during uniaxial elongation [29,30]. Medical devices from polymers, including the piezoelectric ones, are cheap in processing and material costs [31]. Most of the piezoelectric polymers become the right candidates for biomechanical devices, bioelectronics, and biological systems.

Although organic materials exhibit low piezoelectricity compared to inorganic materials, they are the right candidates as functional materials for medical applications. Organic smart materials can be applied in various types of devices, including micro- and nano-scaled medical devices [32,33].

This review provides thorough information on various piezoelectric materials, which can be used in catheter applications, tissue engineering, healthcare monitoring, actuators, and biosensors. We describe inorganic and organic piezoelectric materials and their development, biomedical applications, and properties; we provide a comparison between different inorganic and organic materials and their applications in biodevices summarizing the challenges and trends of piezoelectric materials for medical applications.

## 2. Piezoelectric Materials—Mechanisms of Piezoelectricity

Piezoelectric materials are characterized by crystal or crystal-like structure with three-dimensional order of atoms [34].

### 2.1. Inorganic Materials—Mechanisms of Piezoelectricity

Piezoelectric crystals can occur naturally (e.g., tourmaline) or be synthetic (e.g., lithium niobate). Table 1 shows selected natural and synthetic piezoelectric materials [35].

**Figure 3:**
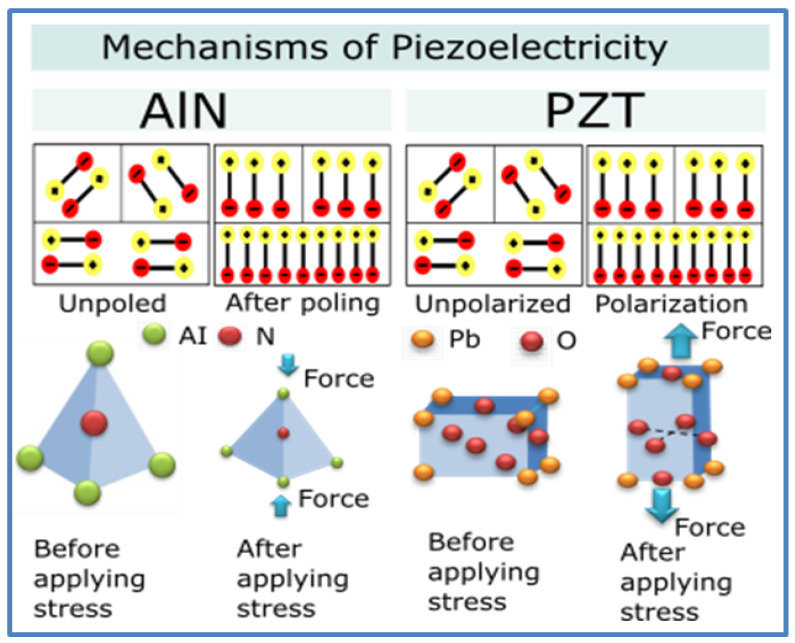
Scheme of mechanisms of piezoelectricity in inorganic materials.

Lead zirconate titanate (PZT) and aluminum nitride (AIN) are two representative materials from the group of synthetic piezoelectric ceramics (Figure 3). The piezoelectric effect, in this case, is described by movements of ions in the crystals under stress, resulting in changes in the balance of ions and the creation of a non-zero crystal dipole moment. For a non-zero net polarization, the atomic structure should be non-centrosymmetric. Several materials with centrosymmetry have symmetry in nonequilibrium or nanoscale conditions, and they become piezoelectric [36].

### 2.2. Organic Materials—Mechanisms of Piezoelectricity

Organic materials are often hierarchically organized with rather low crystallographic symmetry. Thus, the piezoelectric effect in organic materials is the movement of the dipoles in the bulk polymer. It can be the effect of, for example, drawing (stretching) or the action of a high electrical field. The group of piezoelectric materials such as silk and collagen have attracted attention in recent years [37]. Table 2 provides information about piezoelectric coefficients for different piezoelectric inorganic and organic materials.

Polyvinylidene Fluoride (PVDF) and its copolymers are the most investigated materials due to their high piezoelectricity, good chemical resistance, thermal stability, good processability, and mechanical properties, as compared to other piezoelectric materials. Polyvinylidene fluoride (PVDF) can exist in five piezoelectric crystal phases. β-phase has the highest piezoelectricity [48,49,50].

PVDF molecular dipoles CH_2_-CH_2_ can arrange various chain conformations. The strong electric moment is connected with the strongest electro-negativity of fluorine atoms; the crystal can exhibit a non-zero dipole moment [51,52,53]. Such a molecular arrangement appears in the β, γ, and δ phases, the first one showing the strongest dipole moment due to the all-trans conformation. In the case of other chain conformations (TGTG-, T_3_GT_3_G-), parallel dipole moment arrangement, as in the δ and γ phases, respectively, leads to lower polarity; in the case of the same conformations, antiparallel chain dipole arrangement leads to the zero net dipole moment as in the α [54,55].

**Figure 4:**
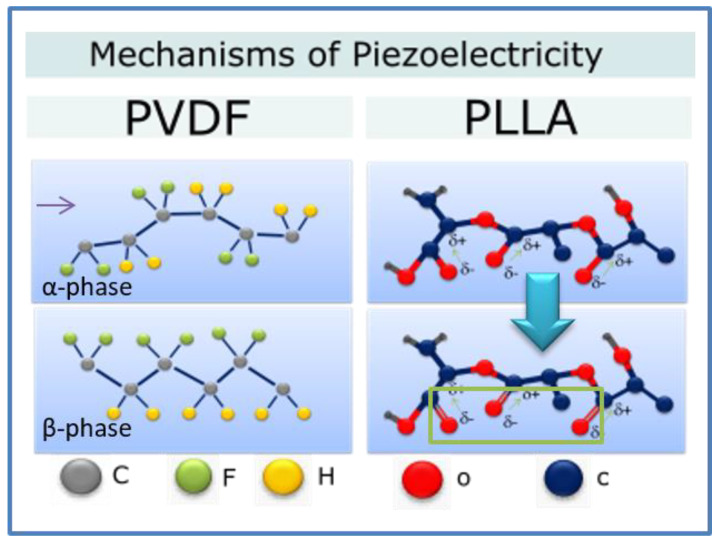
Scheme of mechanisms of piezoelectricity in organic materials.

PLLA is characterized by lower piezoelectricity than inorganic piezoceramics (e.g., PZT); however, in the shape of a film, it has a large piezoelectric shear constant. Figure 4 (right) shows polylactic acid (PLLA) chain in thermodynamic equilibrium corresponding to α-crystal form. Dipoles of CO are statistically distributed in the main chain. α-crystalline form can transform to piezoelectric β-crystal modification during stretching through an arrangement of the dipoles along the stretching direction [56]. The process of fiber electrospinning may also lead to aligning of the CO bonds resulting in piezoelectricity of PLLA. PLLA is a flexible polymer suitable for mobile device applications [57]. This biodegradable and biocompatible polymer is highly potential in future applications such as biosensors and actuators [58,59].

## 3. Inorganic Piezoelectric Materials

### 3.1. Biosensors

Various molecules, microorganisms, or biological structures can be detected using piezoelectric biosensors. Piezoelectric biosensors are made from materials with good acoustic velocity, for instance, a highly sensitive and selective sensor for ethionamide guided by molecular modeling [60], aluminum nitride (AlN) acoustic biosensors providing real-time response and quantified data [61], and highly sensitive AlN biosensor for detecting pesticide residues [62]. Piezoceramic sensors exhibit good reproducibility, good linear response and low detection limit. AlN piezoelectric biosensors can be used to track multiple specific biological reactions, for example, genetic hybridizing, which could provide information on the reaction kinetics. Another application of AlN sensors is the detection of protein-ligand interactions or antigen-antibody binding. Table 3 provides a comparison of inorganic materials for different medical applications.

### 3.2. Energy Harvesting

Energy harvesting is the extraction of electrical energy from various sources and storing it for small autonomous wireless devices. The energy can be harvested from multiple sources such as thermal and mechanical sources. Natural and artificial ambient light and radio frequency can also be harvested. The piezoelectric energy harvesting systems based on piezoelectricity seem the right candidates for use in biomedical electronics [67,68].

Laterally, various groups of piezo-energy harvesters made from materials such as lead magnesium niobate-lead titanate (PMN-PT), zinc oxide (ZnO), lead zirconate titanate (PZT), and barium titanate (BaTiO_3_) have been used to store energy from heartbeats, body movements, and various deformations [69].

For instance, it is reported on a lead zirconate titanate (PZT)-based energy harvester, which can store energy from natural heart movements and other inner organs movements. In this specific device, chromium (Cr) and gold (Au) layers are located on ribbons and act as an electrode. On the bottom of PZT-ribbons titanium (Ti) and platinum (Pt) layers are deposited, which are formed using wet etching. Polydimethylsiloxane (PDMS) stamp is used to seal whole medical harvester devices [70].

Another example may be an energy harvester for the protection of medical devices, in which gold (Au) electrodes are placed on the lead magnesium niobate-lead titanate (PMN-PT) films. Additionally, the epoxy-based photoresist (SU-8) coating layer serves as a protective film in devices and energy harvesters. In vivo tests on the cardiac rat muscles show that electric power could be used to detect heart deformations [71].

Nanogenerators from gallium nitride (GaN) and zinc oxide (ZnO) are piezoelectric and can be used as harvesters the biomechanical energy [72]. Zinc oxide (ZnO) nanowires have attracted much attention because of their various unique properties, e.g., flexibility and application as nanogenerators, sensing, and energy-harvesting devices, and power supplies. Gallium nitride (GaN) nanorods can be used as parts of biomedical devices due to its ability to deform and preserve performance under stress and strain [73,74,75,76].

### 3.3. Tissue Engineering

Piezoelectric actuator-sensor systems for tissue engineering are widely tested and described in the literature. Conventional actuators and sensors for tissue engineering should be occasionally removed from the body because of insufficient flexibility. To avoid the need to remove the device, transient electronic biomedical sensors with high mechanical properties can be applied [77]. Moreover, piezoelectric nanoparticles exposed to ultrasonic waves can change the viability of breast cancer cells [78].

It was shown that lead zirconate titanate (PZT) developed recently in the form of nanoribbons can act as a skin sensor as well as a sensor of deformation of other internal tissues [79]. These medical devices can detect tissue deformations and accumulate information about the mechanical properties of the skin surface. Opposite to the conventional equipment, these sensors can contact underlying topography with the skin surface. Tests on human models revealed the great need for non-invasive devices to define the skin’s mechanical properties [80]. So far, PZT nanoribbons were also tested on lung-stimulated respiration; thus, further investigations on humans are necessary [81].

## 4. Organic Piezoelectric Materials

### 4.1. Biosensors

Organic piezoelectric materials have different properties compared to inorganic piezoelectric materials. Thanks to good flexibility and mechanical properties, they are susceptible to external stimuli, crucial for various medical device applications [82]. In the case of medical sensors, which are placed near internal organs, e.g., a heart, good flexibility is the main feature allowing them to use in medicine [83,84,85,86,87,88].

Consequently, different layers for sensors made from textiles [89,90,91,92], hydrogels and soft polymers [93,94,95], elastomers [96,97,98], nanofiber layers [99,100,101], or thin polymer layers [102,103,104] are used. Additional piezoelectric, piezoresistive, triboelectric, thermoelectric properties of these piezoelectric materials make them good candidates for smart sensing applications [105,106,107]. The characterization of organic piezoelectric sensors is provided in Table 4.

Smart medical sensors electrospun from polyaniline and polyvinylidene fluoride (PANI/PVDF) were developed to detect the tissue tension [115]. Compared with electrospun polyaniline and polyvinylidene fluoride (PANI/PVDF) mats, smart sensors can detect 11% strain and possess linear response up to 85% strain. Other groups of electrospun sensors have excellent sensing performance and high stretching in medical applications [116,117]. Nevertheless, the medical sensor response is insufficient regarding horizontal polarization structure. To detect normal force efficiently, vertically oriented polyvinylidene fluoride (PVDF) fibers have been fabricated to obtain new types of pressure sensors [118]. Furthermore, with other nanowire-shaped piezoelectric sensors from highly aligned polyvinylidene fluoride-trifluoroethylene P(VDF–TrFE) with excellent sensitivity (458.2 mVN^−1^), basic life functions such as breath or pulse can be monitored [119].

Another group of piezoelectric sensors is being developed to monitor the incessant changes in the environment. Sensors in the shape of a film made from polyvinylidene fluoride (PVDF) and reduced graphene (rGO) can determine many external thermodynamic parameters, such as temperature or pressure. Several tests conducted by researchers resulted in the improvement of the sensors’ properties [120].

Many groups work on the next generation of piezoelectric sensors for advanced biomolecules detecting [121]. Biosensors work on the principle of specific reactions to detect molecules, enzymes, or antibodies [122]. Moreover, some medical devices can determine the release of nitric oxide (NO) [123] or other molecules [124]. A biosensor based on PVDF films can detect nucleic acid, which is important for a disease diagnosis [125]. As well, piezoelectric biosensors can capture the pathogens [126]. In some cases, the placement of decomposable polylactic acid (PLLA) sensors inside the human body causes faster wound healing. An excellent example of a PLLA sensor is the photolithographic sensor used to determine specific physiological forces [127]. This PLLA sensor is made using an annealing or stretching process at 90 °C at 8 h. The film of size 3 mm × 15 mm was cut at 45° angle to fabricate the sensor. The ease of manufacture makes it very attractive. Table 5 provides a comparison of some organic materials for different medical applications.

### 4.2. Catheter Applications

Catheters are essential medical devices. For example, the catheter can be used as a biomaterial for drug delivery to collect information during surgeries or as stents or prostheses [135,136].

Polyvinylidene fluoride (PVDF) is the right material for sensing elements in biomedical catheters because of its remarkable piezoelectric properties [137,138,139]. Electrospun PVDF sensors have been used as part of the catheter to minimize complications after surgeries [140] and to define the real-time flow in a medical catheter [141].

Medical sensors from PVDF copolymers, P(VDF–TrFE), fabricated in the shape of films, are used for intravascular measurements. Biocompatible catheters with good mechanical properties exhibit great adhesion. Piezoelectric polyvinylidene fluoride (PVDF) (shell) core-shell nanofibers with the addition of polyethylenedioxythiophene (PEDOT) (core) can be used as an electrode, which is a part of catheter devices. Highly aligned fibers were manufactured to boost the device. This electrode provides information about the sensitivity of the structures [142].

### 4.3. Healthcare Monitoring

Piezoelectric sensors for healthcare monitoring can measure, in real time, the physiological functions of human organisms via dynamic measurements. For disease diagnosis, non-invasive biosensors transfer the data from, e.g., interstitial fluids, sweat, tears or saliva [143].

Furthermore, electronic skin (e-skin) used in healthcare monitoring systems have been developed. Thanks to its remarkable properties, e-skin has been used in various medical applications, such as wearable sensors and disease diagnosis [144]. Pressure biosensors can imitate chameleons’ skin allowing the color change in some devices [145]. For example, another pressure sensor can interact with e-skin [146]. Composite material from polyvinylidene fluoride (PVDF) and reduced graphene was used to fabricate piezoelectric artificial skin. PVDF/GO films with high conductivity were cast and annealed to receive high content of β-phase and used to determine multiple stimuli, including temperature or pressure. Solution casting was provided in 50 °C to crystallize polar phases in PVDF and then annealed at 160 °C. [147].

PVDF/Au biosensors are used for different pressure sensing applications. Gold films perform as electrodes, and a silicon substrate is used to enhance flexibility. These sensors can be placed in different places in the human organism for healthcare monitoring and sensing, for example, to measure respiration levels or monitor various physiological systems. To help patients with paralysis, piezoelectric sensors can measure muscle movements [148,149,150].

Noteworthy are natural polymer-based healthcare monitoring systems made from collagen [151,152], and silk [153,154]. Another group of flexible medical devices worth mentioning is made from fish skin; they are used as individualized healthcare monitoring systems [155].

### 4.4. Actuators in Tissue Engineering and Devices

Medical actuators are an important group of piezoelectric devices used for motion control. The designs of piezoelectric actuators are an effect of very advanced research [156].

However, piezoelectric forces are often very weak, and piezoelectric actuators have been adapted to various applications characterized by small displacement. Piezoelectric tissue actuators are flexible and biocompatible and can be used to promote tissue regeneration [157]. Certain smart actuators are reported to have the potential to replace a damaged organ in human organisms [158,159,160,161]. Textile actuators can mimic the motion of the human body [162,163,164,165], whereas shape-memory polymers (SMP) and shape-memory alloys (SMA) can be activated by stimulation [166,167,168,169,170].

In a series of papers, it is reported on PLLA tweezers for the treatment of thrombosis. In the form of biodegradable fibers, the tweezers, inserted into a blood vessel, reveal the high potential to grasp silica compounds. Fibers were produced by jet spinning and treated by alternating current (AC) voltage. Thanks to the above-mentioned properties and high sensitivity, these piezoelectric tweezers seem to be good candidates for nanomedicine and tissue engineering applications [171,172,173,174].

Piezoelectric materials can be produced using nanocomposites containing oriented polymer fibers, which are responsible for actuation and sensing functions. Shells from polyvinylidene fluoride/carbon nanotubes (PVDF/CNT) are reported to have better efficiency than standard devices [175]. Polyvinylidene fluoride (PVDF)-based actuators were used in bone tissue engineering applications. Osteoblasts were incubated on the piezoelectric ground. It was found that dynamic conditions improve the cell proliferation [176,177].

Compact sensor-actuator devices with the addition of silver nanoparticles can be used for strain sensing. These devices can be also used for transforming vibration into electrical energy and electrical energy to mechanical energy, for producing signals and ultrasonic energy, and drilling equipment. Piezoelectric actuators have many industrial applications, such as in musical instruments’ parts, phones, or complex music systems. In medicine, piezoelectric actuators are widely investigated in endoscope lenses, small pumps, and other applications [178].

Furthermore, 3D fibrous piezoelectric scaffolds can be used with stimulation during in-vitro tests and to support osteogenic differentiation. However, piezoelectric materials with small voltage output support chondrogenic differentiation. Finally, the test shows that electromechanical stimulation improves chondrogenic differentiation. Electromechanical actuation is higher than under mechanical actuation [179].

It is reported that the parameters of the electrospinning process can substantially affect the properties of the nanofiber actuators. Optimization of the process allows the fabrication of smart PVDF bio-actuators for energy harvesting. Results showed fibroblasts developed perfectly along the fiber direction [180]. Such smart scaffolds, due to its electric effect, can be applied in neural tissue engineering for nervous system regeneration [181]. Smart PVDF-based scaffolds, in the presence of ultrasonic waves, display neurite proliferation in PC-12 cells [182]. In another work, piezoelectric films were subjected to high-intensity ultrawaves (>1 W/cm^2^). In these experiments, various physical effects, such as radiation force, affected cells during ultrasonication [183].

Another nanocomposite system based on PVDF with the addition of barium titanate nanoparticles has been tested in the presence of ultrasonic waves. The material was fabricated using the electrospinning process. Results showed that stimulation promoted the viability of the cells [184].

Recently, the newest tests provided information on the effect of the application of smart PVDF scaffolds in promoting cell communication. Electro-active cardiomyocytes were tested during stimulation. Electrical stimulation affected the in-vitro cells differentiation and proliferation [185].

The effects of PVDF copolymerization on the stem cells were investigated using polycaprolactone (PCL) films with PIEZO P(VDF–TrFE) layer [186], proving the improvement of the cell viability; however, further investigations are still necessary. Fibers were investigated as PCL spin-coated microfibers. Piezoresponse force microscopy (PFM) showed a high value of piezoelectric constant d_14_ = 11.1 pmV^−1^. Finally, this type of scaffolds promotes rat and human cells proliferation [187].

## 5. Conclusion and Future Outlook

We have reviewed various applications of smart piezoelectric materials in medical devices and electronics, such as earphones, telephones, and complex electrical systems. It may be clearly seen that piezoelectric materials constitute a group of modern materials. They can effectively transform energy from mechanical to electrical and vice versa, they can be biocompatible after particular additional treatment giving them the status of piezoelectric biomaterials with an excellent perspective for application in many medical devices.

We have summarized various principle inorganic and organic piezoelectric materials used in sensors, actuators, catheters, healthcare monitoring, and tissue stimulators. We presented a comparison between different inorganic and organic piezoelectric materials.

Although inorganic piezoelectric materials have been long and extensively explored, organic biomaterials have emerged as a relatively new group of modern materials offering unique properties, especially in sensing applications due to their flexibility. Therefore, piezoelectric materials are widely investigated in applications such as actuators, sensors, and medical devices.

By describing the examples of the challenges, we hope to bring a greater understanding of how vital to the industry, mostly medical, piezoelectricity is, and the opportunities in future development, including brain machines, neurostimulators, smart interfaces, or in the auto-control smart systems.

## Figures and Tables

**Table 1:** Selected natural and synthetic piezoelectric materials.

	Material	Chemical Formula
Natural	α-quartz	SiO_2_
	β-quartz	SiO_2_
	Tourmaline	(Na, Ca)(Mg, Fe)_3_B_3_Al_6_Si_6_(O, OH, F)_31_
Synthetic	CGG	Ca_3_Ga_2_Ge_4_O_14_
	Lithium niobate	LiNbO_3_
	Lithium tantalate	LiTaO_3_
	Aluminum nitride	AlN
	Lead zirconate titanate	PZT

**Table 2:** Piezoelectric coefficients for different piezoelectric inorganic and organic materials.

	Material	Type	Piezoelectric Constants		Refs.
			d_33_ (pC/N)	d_31_ (pC/N)	
Inorganic	PMN-PT	Single Crystal	2000–3000	-	[38]
	Quartz		2.3	−0.67	[39]
	ZnO	Crystal	6–13	−5	[40]
	GaN		2–4	−1.5	[41]
	AIN	Ceramic	3–6	−2	[42]
	PZT-5H		593	−274	[43]
	BaTiO_3_		190	−78	[44]
	LiNbO_3_		16	−1	[45]
Organic	PVDF	Polymer	−33	23	[46]
	PLLA		6–12	-	[47]

**Table 3:** Inorganic piezoelectric sensors for different medical applications.

	Material	Applications	Device Characteristics	Refs.
Inorganic	(Na_0.5_, K_0.5_) NbO_3_ (NKN) films	Mechano-electrical sensor	10 Hz resonance	[63]
	AlN	Monitoring of respiration and heartbeat	Tested over 0.1–10 Hz	[64]
	PZT	Eye fatigue	-	[65]
	Ceramics sensors—PZT	Vision correction	Sensitivity 0.1 × 10^−2^ N to 5 × 10^−2^ N 0.01^–5^ Hz	[66]

**Table 4:** Organic piezoelectric sensors with characteristic properties.

Structures	Stability	Sensitivity	Defect Limit	Sensing	Refs.
PVDF/rGO nanohemispheres	5000	35 kPa^−1^	0.6 Pa	0.6 Pa–49.5 kPa	[108]
PVDF/BaTiO_3_ nanopillars	12,000	0.0264 kPa^−1^	-	50–600 kPa	[109]
PVDF/PANI nanofibers	10,000	1.84 kPa	-	0–110% strain	[110]
P(VDF–TrFE) nanofibers	-	1.1 kPa^−1^	0.1 Pa	0.4–2 kPa	[111]
P(VDF–TrFE) nanowires	36,000	0.046 kPa^−1^	-	-	[112]
P(VDF–TrFE) nanopyramids	5000	0.005 N^−1^	-	-	[113]
P(VDF–TrFE)/GO nanofibers	100,000	15.6 kPa^−1^	1.2 Pa	-	[114]

**Table 5:** Comparison of organic piezoelectric sensors for different medical applications.

	Structures	Applications	Device Characteristics	Refs.
Organic	Prawn cell	Wrist pulse	100 Hz–10 MHz range	[128]
	Fish gelatin	Joint movement Cord movement Pulse	d33–20 pm/V 108,000 cycles	[129]
	PVDF	Human voice detection Hand motion Breathing	50–1000 Hz range	[130]
	PVDF	Wrist pulse Pressure pulse	-	[131]
	PVDF	Heartbeat and respiration detection	Tested 0.1–2 Hz	[132]
	PVDF	Food detection by swallowing pattern	Limit of detection: 1 Hz Tested over 1–5 Hz	[133]
	Poly-L-lactic acid	Pressures: brain, eye	108,000 cycles	[134]
